# Internet-Based Approaches to Building Stakeholder Networks for Conservation and Natural Resource Management

**DOI:** 10.1007/s00267-015-0624-8

**Published:** 2015-10-27

**Authors:** B. J. Kreakie, K. C. Hychka, J. A. Belaire, E. Minor, H. A. Walker

**Affiliations:** Atlantic Ecology Division, Office of Research and Development, U.S. Environmental Protection Agency, 27 Tarzwell Drive, Narragansett, RI 02882 USA; St. Edward’s University, 805 North Capital of Texas Highway, Austin, TX 78746 USA; Department of Biological Sciences, University of Illinois Chicago, 845 W. Taylor Street, Chicago, IL 60607 USA

**Keywords:** Cybermetrics, Hyperlink, Relatedness, Social network analysis, Stakeholder

## Abstract

Social network analysis (SNA) is based on a conceptual network representation of social interactions and is an invaluable tool for conservation professionals to increase collaboration, improve information flow, and increase efficiency. We present two approaches to constructing internet-based social networks, and use an existing traditional (survey-based) case study to illustrate in a familiar context the deviations in methods and results. Internet-based approaches to SNA offer a means to overcome institutional hurdles to conducting survey-based SNA, provide unique insight into an institution’s web presences, allow for easy snowballing (iterative process that incorporates new nodes in the network), and afford monitoring of social networks through time. The internet-based approaches differ in link definition: hyperlink is based on links on a website that redirect to a different website and relatedness links are based on a Google’s “relatedness” operator that identifies pages “similar” to a URL. All networks were initiated with the same start nodes [members of a conservation alliance for the Calumet region around Chicago (*n* = 130)], but the resulting networks vary drastically from one another. Interpretation of the resulting networks is highly contingent upon how the links were defined.

## Introduction

The work load for conservation professionals is ever growing, as is our sense of urgency and necessity. However, the extent of conservation achieved is severely limited by financial resources. Social network analysis (SNA) is an invaluable tool, which could help maximize limited financial resources to accomplish more of the conservation work load (Bodin and Crona 2009; Bodin and Prell 2011). SNA is based on a conceptual network representation of social interactions, in which the actors are represented by nodes and links (or edges in graph theory terminology) are established between nodes depending on specific types of social interactions.

SNAs have been used by conservation organizations to identify key individuals who can or do perform certain functions (Kimmel and Hull 2012) and by researchers to identify characteristics of the network that relate to adaptive management (Bodin et al. 2006). SNA can provide information about stakeholders’ relative influence in the network, which can help identify opportunities for and barriers to collaboration, information flow, and efficiency—all characteristics that contribute to adaptive capacity (Armitage 2005) and social capital Pretty (2003). Collaboration, specifically, can provide a cost-effective means for organizations to fill deficiencies and to avoid redundant efforts (Keough and Blahna 2006). SNA can identify a pool of potential collaborators operating at different spatial scales, locations, and levels of governance, and working on different conservation issues (Cohen 2011; Cohen et al. 2012; Crona and Hubacek 2010; Vance-Borland and Holley 2011). Identifying bridging organizations, which have numerous contacts and link otherwise disconnected parts of the network, could increase information flow and reduce the amount of outreach efforts for a relatively disconnected node (Connolly et al. 2012). While conservation biologists are often criticized for not communicating with managers (Arlettaz et al. 2010; Knight et al. 2008), a stakeholder network could quickly identify the resource agencies best able to implement new management strategies. Even though SNA can be a valuable tool in natural resource conservation, these methods have not been fully utilized by practitioners for several reasons.

Traditional approaches to constructing stakeholder networks generally rely on social surveys (Newman 2003; Wasserman and Faust 1994). Researchers give a survey to stakeholders, identified from lists of attendees at a conference or benefit, membership of boards or email list-servs, or best professional judgment (Durland and Fredericks 2005), to identify which individuals or organizations with whom they have contact. The network is grown through snowballing which is an iterative process for adding new nodes to the network by surveying individuals or organizations added to the network through the first round of surveys. These traditional approaches present several major hurdles which may prohibit interested individuals from conducting such analysis. One difficulty is poor survey response rate. Baruch and Holton (2008) conducted a meta-analysis of 490 publications and found an average survey response rate of 52.7 % (±20.4 %) for individuals and 35.7 % (±18.8 %) for organizations. The response rate for email or web-based surveys is even lower (Cook et al. 2000; Sheehan 2001). Additionally, survey-based research may be legislatively or time prohibitive for research scientists working for the federal government (Presser and McCulloch 2011).

Networks derived from cybermetrics, which quantify the structure and information of the Internet (Björneborn 2004; Björneborn and Ingwersen 2004), can overcome some of the shortcomings and hurdles of traditional survey-based methods. In the early 1980s, Freeman (1984) examined 50 scientists using a primitive electronic communication system and concluded that this form of communication changed how researchers established connections and how their social networks were structured. Since Freeman’s work, the field has exploded and researchers have analyzed networks derived from cybermetrics for a range of systems, including the evolution of social networks over time based on emails between university students (Kossinets and Watts 2006), and a 40-year gap between the discovery and delivery of findings about second-hand smoke based on citation analysis of public health publications (Harris et al. 2009).

Specifically, we present two Internet-based methods for defining links in a SNA. The first method is based on hyperlinks between organizations’ websites. This method assumes some level of organizational interaction if the organization inserts a hyperlink from their page to another organization’s page. The second method is based on a search engine’s ranking of website relatedness. This method operates on the assumption that websites are connected based on how Internet traffic is directed around the Internet. For completeness, we also created networks based on combining the results of these two methods of defining links. These internet-based approaches build the network through fundamentally different approaches than a traditional approach—a traditional approach actively gathers information about the type and quality of relationships between individuals or organizations. While the hyperlink approach passively collects existing web-based information about explicit partnerships or stated affinities between organizations, the relatedness approach passively collects information about organizational affinities, but not necessarily affiliations between nodes. Therefore, while we do not expect the Internet-based methods to provide a one-to-one replacement for the survey-based methods, we do anticipate that these methods will provide important information regarding the social network structure along with increased understanding of Internet position.

We propose that information gleaned solely from the Internet can provide a rapidly accessible, inexpensive source for building social networks for natural resource and conservation professionals. Further, we suggest that analysis of internet-based social networks can help organizations better position themselves on the Internet and easily monitor changes in the network through time. Specifically, we present two unique approaches to constructing internet-based social networks, and contrast the results from these approaches to a more traditional approach of social network construction. Ultimately, we hope that the work presented here will provide conservation professionals with some guidance on methodology and interpretation of social networks generated from internet-based information.

## Methods

To understand the implications of internet-based approaches to network building, we used an existing survey-based network (the “Calumet Network”; Belaire et al. 2011) to guide the construction of the internet-based networks. This allowed us to illustrate how the web-based methods might be used to complement traditional methods of SNA.

### The Calumet Network

Belaire et al. (2011) studied relationships among environmental groups working in the Calumet region near Chicago, Illinois (USA). Relationships were identified based on social surveys; an email with a link to an online survey was sent to each individual who registered for a conference held in spring 2010 that was intended to bring together all organizations working on conservation, restoration, and remediation in the region. Survey participants were asked to identify organizations on the list of conference registrants with which they had contact (defined as friendship, collaboration, receiving funds, serving on a committee, or exchanging ideas). Participants were also asked to list any contacts not listed explicitly on the survey (i.e., contacts that had not registered for the conference). Responses from multiple individuals from the same organization were aggregated to the organizational level. These additional organizations were included in the final network, but were not asked to complete a survey. Nodes in this network were organizations that completed the survey and any organization that they added (see Table 1 for relevant term definitions). Links were defined through contacts explicitly identified by the organizations.

**Table 1:** Definitions of relevant social network terms

Term	Definition	Reference
Node	Fundamental unit of a network. For our research, this unit is typically an organization or may also be thought of as a website	Newman (2003)
Start nodes	List of nodes selected a priori from which the networks were built based on link definitions described below. All networks created were initialized with the same start nodes. However, the final nodes will differ based on researchers’ rules for growing the network or node snowballing	Newman (2003)
Link	Links or connections between nodes. The networks in this research were created by varying the way in which links are defined: hyperlink, relatedness, survey-based (traditional), or combined approaches	Newman (2003)
Hyperlink network	Network constructed by following electronic links provided on the start node websites that automatically move the browser to a new web address	Defined by the work presented here
Relatedness network	Network constructed by identifying websites related websites to the start nodes based on a measure of the similarity between two websites derived from a proprietary algorithm developed by Google	Google Support (2012)
Traditional network	Network constructed by surveying start nodes. Starts nodes are asked to identify missing nodes and their links in the network. Depending on the methods, nodes may be snowballed into the network and also asked to complete survey	Belaire et al. (2011)
Combined network	Network constructed by merging all unique nodes and links from the hyperlink and relatedness networks	Defined by the work presented here
Focal network	Reduced network comprised only of nodes with degree greater than or equal to two. Each method of network construction has a corresponding focal network	Defined by the work presented here
Metrics		
Average degree	Average number of links per node	Belaire et al. (2011), Vance-Borland and Holley (2011)
Average path length	Average number of steps between any two nodes in a network	Vance-Borland and Holley (2011)
Betweenness	How much each node contributes to minimizing the distance between nodes in the network; variation in the number of times nodes lie on the path between two other nodes (1 indicates all links pass through a single node)	Vance-Borland and Holley (2011), Bodin et al. (2006)
Diameter	Maximum number of steps between any two nodes in the network	Vance-Borland and Holley (2011), Bodin et al. (2006)
Link density	Number of links divided by the number of possible links (ranges from 0 to 1)	Belaire et al. (2011), Vance-Borland and Holley (2011), Bodin et al. (2006)
Modularity	How divided the network is based on predefined communities; in this case, we used walk trap (based on random walks) and link betweenness (prunes out links with highest betweenness to leave the portions of the network they connect) to define communities	Newman and Girvan (2004)
Reciprocity	Proportion of links that are bi-directional (claimed by both organizations)	Vance-Borland and Holley (2011)

### Node Definition

The Calumet study began with a roster of organizations, which had registered for a local conservation conference. We also used these organizations as start nodes for the internet-based networks, although some organizations were omitted from analysis because they did not have a website. We also omitted, due to incomplete surveys, an organization that Belaire omitted from their final results. We restricted our analysis to the root web address (or the main organization). For example, the original Calumet study included two separate branches of a federal government agency, which we reduced to a single node. We revised the Calumet network to reflect these reductions so that all networks included in this research began with the same 130 organizations.

We classified nodes into ten categories based on organization type: advocacy, college, commercial, museum/library, industry, information, K-12 schools, and government (local, state, and federal). For our research purposes, we added the information category not in the original Calumet study. We felt it was an important addition due to the nature of the internet-based approaches. There are numerous websites that present information, but these sites would not be technically classified as news sources.

### Link Definition

#### Hyperlink

Hyperlinks are clickable text or images on a website that direct the user to a new page or different website. Hyperlinks are used to establish directional links between the websites on the Internet (Weare and Lin 2000). Code was written to explore the websites’ HTML code and gather all hyperlinks coded on the start node’s home page or secondary pages titled “partners” or “links.” We selected these terms, because it is common for websites to have either a “link” or “partner” pages linked directly from the homepage. These links are directional in nature. If one web site has a hyperlink to another organization’s website, there is not necessarily a reciprocal link in the opposite direction. All processing was conducted in R (version 2.13.1) on July 17, 2012.

#### Relatedness Links

We used a second method for defining links based on Google’s “relatedness” measure. This is a proprietary method used by Google to identify pages “similar” to a URL based on link structures and other characteristics (Google Support 2012). Searching with the “relatedness” operator returns an ordered list of pages that are most similar to the target site. We chose to pursue this avenue of network generation, because 83 % of adults in the United States use Google as their primary search engine (Purcell et al. 2012), and the algorithms are based on careful understanding and monitoring of the Internet, far beyond the capacity of the authors to compile or craft on their own. Capturing and growing the network using the relatedness search was automated with a series of R scripts that iteratively returned a list of the ten most similar websites. The scripts for the relatedness searches were run on July 20 through 24, 2012 in R (version 2.14.0).

#### Snowballing Procedure

The networks were grown by identifying all outbound links for each start node using two link definition methods described above. We collapsed web addresses to the organizational level by truncating the web addresses to the first “/” after omitting the hypertext transfer protocol portion of the address. Often the reduction to the root web address was sufficient. However, we manually recoded some organizations that had completely different web addresses for different portions of their organization. Also, self-referencing or repeated links were omitted.

Unlike the Calumet study, the internet-based networks were snowballed multiple times. Once the outbound links from a web site were found, we calculated the degree (number of links connected to the node) for all nodes and plotted the distribution. Any node that was not a start node and was above a degree threshold (*n* = 2) was snowballed into the list of start nodes and had outbound links defined. For each of the internet-based methods of link definition, the snowballing procedure was run independently. Therefore, the composition of each network could vary greatly aside from the original start nodes.

### Sensitivity Analysis

We conducted a sensitivity analysis to determine how the final network was affected by the initial list of start nodes. We initialized each iteration of the sensitivity analysis with a random subsample of the start nodes, starting with 5 % of the complete list and incrementally increasing at 5 % intervals to 95 %. We then grew the networks according to the rules described above for both hyperlink and relatedness link definitions and recorded the percentage of the full network that was acquired. This process was repeated 100 times for each percentage of start nodes.

### Network Analysis

We assumed that resource managers may want to use both internet-based methods in order to create a more comprehensive list of stakeholders. As illustration of this approach, we included a combined network. This network incorporated the nodes and links from the hyperlink and relatedness networks, and will be henceforth referred as the combined network.

We used several network metrics that relate to adaptive management of natural resources (Bodin et al. 2006; Vance-Borland and Holley 2011) or were used to characterize the original Calumet network (Belaire et al. 2011): average degree, average path length, betweenness, diameter, link density, modularity, and reciprocity (Table 1). We calculated the network metrics in R 2.14.0 either manually or using the R package iGraph 0.6 (Csárdi and Nepusz 2006).

## Results

All networks presented here were initialized with the same 130 start nodes. However, several of the start nodes were dropped depending on the method’s link definition. The hyperlink approach retained 106 start nodes in the final network, because some websites either did not have any hyperlinks or only had hyperlinks to other portions of their own website (i.e., self-loops). The relatedness method retained 117 start nodes, because the Google relatedness search returned no related websites for the other sites.

**Fig. 1:**
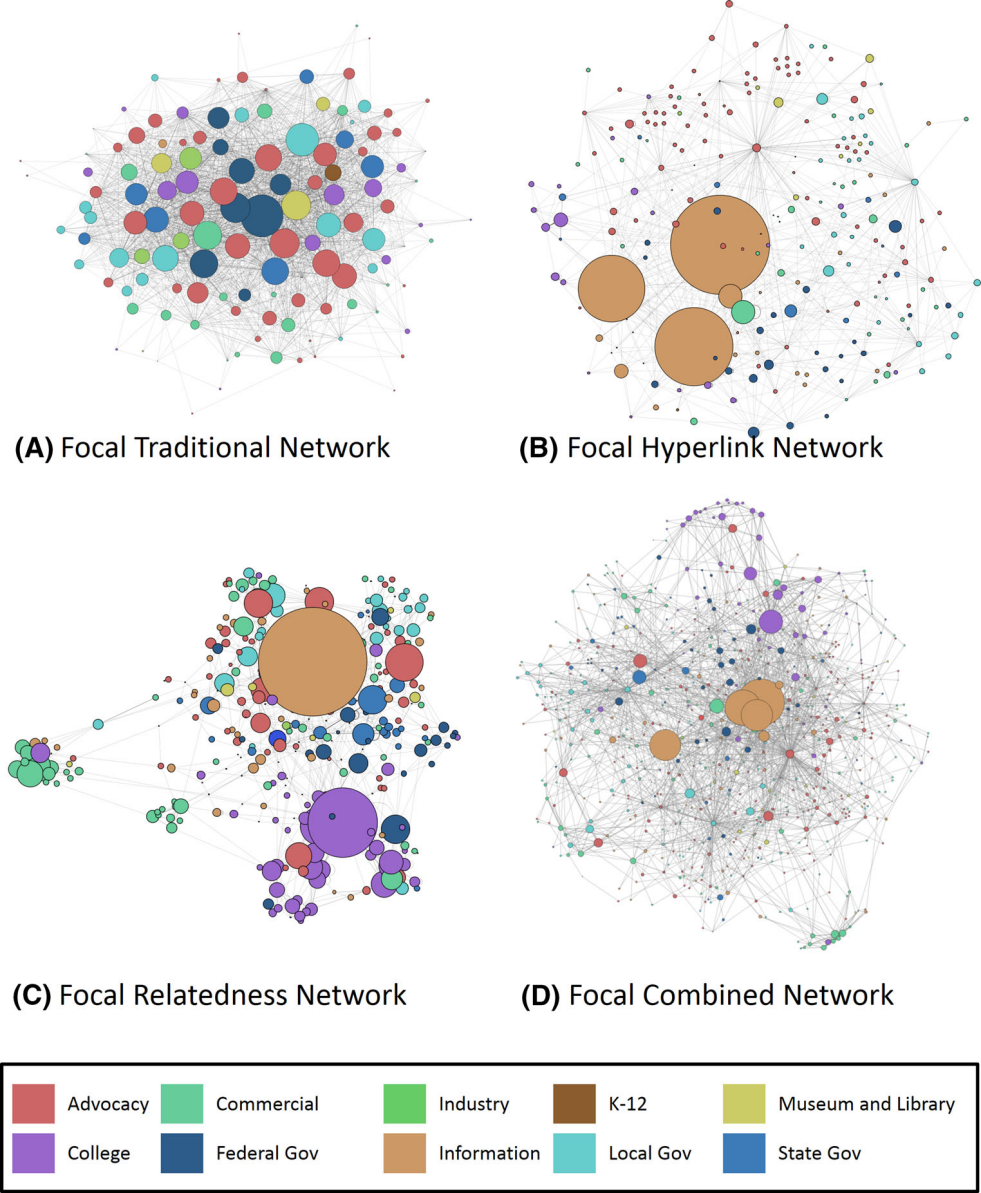
Network diagrams including only nodes with a total degree of two or higher. The nodes are color-coded according to organization type. The size of the node is scaled to its in-degree. **a** Traditional focal network: based on social survey link definition (results of Belaire et al. 2011). **b** Hyperlink focal network: based on hyperlink definition. **c** Relatedness focal network: based on Google’s relatedness link definition. **d** Combined focal network: network developed from using the results of both the hyperlink and relatedness networks

The hyperlink and relatedness methods produced networks with a large number of nodes with a single link. To focus our attention on the most influential portion of the network, we created subsets of each network that omitted all nodes with only one link (referred to hereafter as the “focal” version of the network; Fig. 1). This truncation of one degree nodes occurred after all snowballing iterations, and after adding final full hyperlink and full relatedness networks for the full combined network. Thus, the focal combined network was not simply additive of the focal hyperlink and focal relatedness networks.

There were striking differences between the networks (Fig. 1) which can be quantitatively explored with a series of metrics (Table 2). The traditional network had a higher average degree than all the other methods. The focal traditional network had a 20.8 average degree compared to an average degree of approximately 3 for the other focal networks. This high connectedness is also reflected in the high link density of the traditional network (0.18 and 0.16 for focal) and lower connectedness for all other focal networks (0.01). The high relative connectedness of the nodes in the traditional network resulted in relative decrease in network distances between nodes. The diameter for the traditional network was 4, and average path length was 1.83, whereas the focal hyperlink and relatedness networks had diameters of 6 and 14 and the average path lengths of 2.4 and 5.1, respectively. Reciprocity was highest for the traditional network (46 %), fairly high for the focal relatedness network (29 %), and considerably lower for the focal hyperlink network (4 %). To compare networks with more similar bounding, Table 2 also presents the network metrics for the base focal networks (no snowballing). Notably, even though the internet-based networks grew considerably in number of nodes and links, the general construction of the networks, in terms of network metrics and the proportion of nodes by group, remained unchanged through all snowballing iterations.

**Table 2:** Summary of network characteristics

	Combined	Hyperlink	Relatedness	Traditional
Base				
Number of nodes	287	165	173	127
Number of links	915	524	384	2636
Diameter	12	6	8	4
Average degree	3.19	3.18	2.22	20.76
Link density	0.01	0.02	0.01	0.16
Reciprocity	0.07	0.03	0.09	0.46
Average path length	4.40	2.34	3.24	1.83
Modularity	0.34	0.31	0.61	0.11
Fully snowballed				
Number of nodes	661	230	337	–
Number of links	2318	712	1116	–
Diameter	11	6	14	–
Average degree	3.51	3.1	3.31	–
Link density	0.01	0.01	0.01	–
Reciprocity	0.16	0.04	0.29	–
Average path length	4.69	2.44	5.06	–
Modularity	0.46	0.37	0.62	–

The top portion presents the metrics for the base networks (before snowballing), while the bottom proportion presents the metrics after all snowballing was completed

We calculated several node-level metrics (degree, betweenness, and constraint) to identify the important nodes in each network (See Table 1 for metric definitions). The five nodes with the highest degree were completely different between the internet-based approaches and the traditional network. Two nodes had the highest degree in both the relatedness and hyperlink network; both were universities. There were no commonalities between the different approaches for nodes with highest betweenness and constraint. Essentially, each network approach generated completely new lists of most impactful nodes.

The distribution of nodes among different organization types varied between the networks (Table 3). The traditional network had very few nodes classified as informational organizations, while the focal relatedness and hyperlink networks had 12 and 9 %, respectively. Another difference between the networks was that the relatedness network had a lower proportion of advocacy organizations than the others.

**Table 3:** Summary of node distribution across type of organizations

		Combined	Hyperlink	Relatedness	Traditional	
		Focal	Focal	Focal	Focal	Full
Number of nodes		661	230	337	127	153
Number of links		2318	712	1116	2636	4140
Proportion of nodes						
Advocacy		0.31	0.36	0.23	0.38	0.39
College		0.10	0.10	0.16	0.13	0.15
Commercial		0.20	0.13	0.20	0.16	0.15
Museum/library		0.03	0.03	0.03	0.02	0.02
Industry		0.00	0.01	0.01	0.03	0.04
Information		0.11	0.09	0.12	0.01	0.01
K-12		0.00	0.00	0.01	0.01	0.01
Local government		0.14	0.13	0.11	0.14	0.14
State government		0.05	0.04	0.08	0.05	0.05
Federal government		0.06	0.11	0.07	0.06	0.05

Not only did the networks vary in structure, they varied substantially in the link and node composition. This can be illustrated by examining the deviations of the final focal hyperlink and relatedness from the traditional network. The focal relatedness, hyperlink, and combined networks captured 60, 67, and 83 % of the nodes in the traditional network, respectively. Links had far less agreement between methods, with all three internet-based networks capturing much <1 % of the links in the traditional network.

**Fig. 2:**
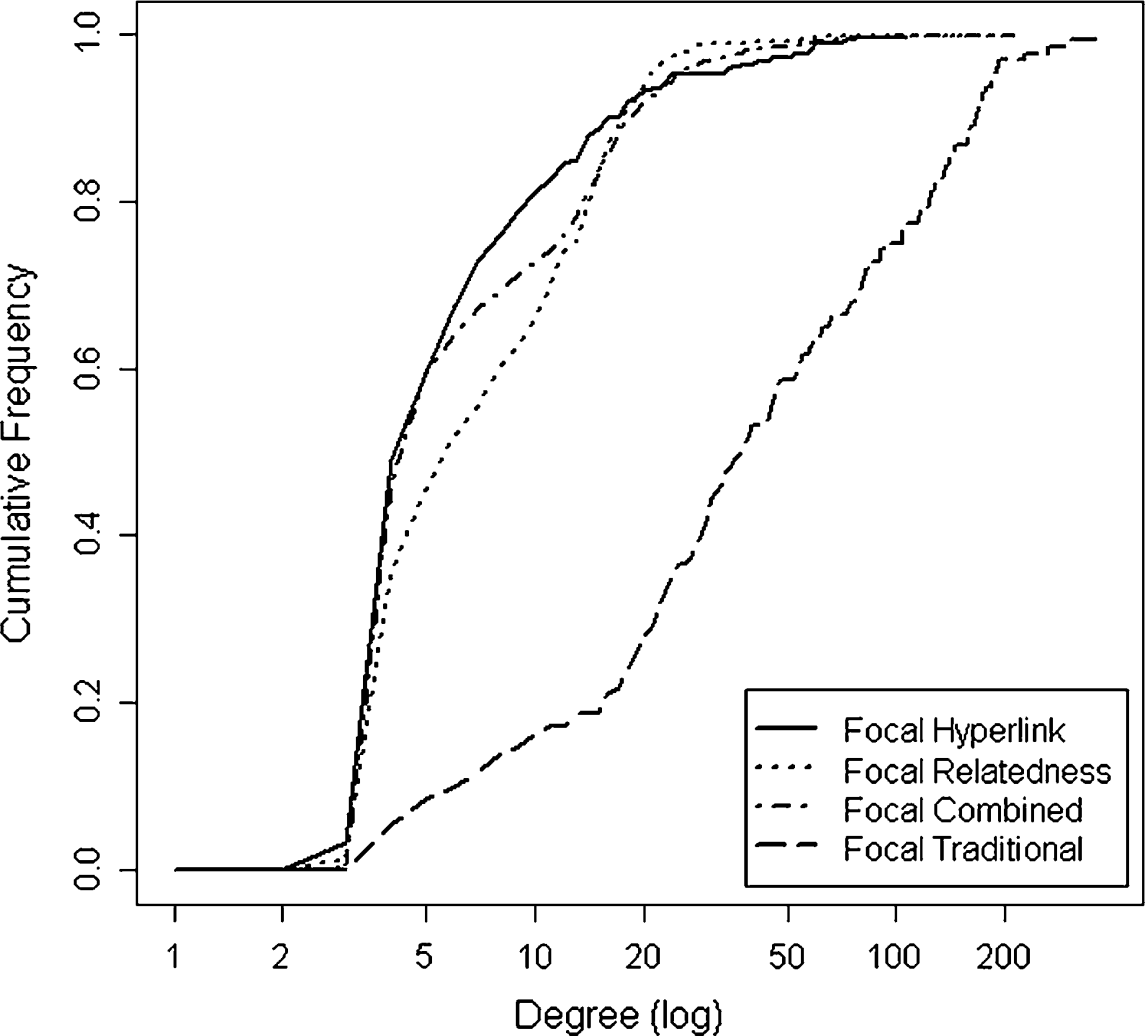
Plot of cumulative frequency of degree for each focal network (note log scale)

The focal hyperlink network had very few nodes with high degrees and a large number of nodes with low degrees (Fig. 2) (Aberer et al. 2004; Broder et al. 2000). The focal relatedness network had a very similar distribution with a somewhat higher number of nodes with intermediate degree values. The focal traditional network had relatively smooth distribution with very few nodes having a low degree; half the nodes in the network had 40 or more connections.

**Fig. 3:**
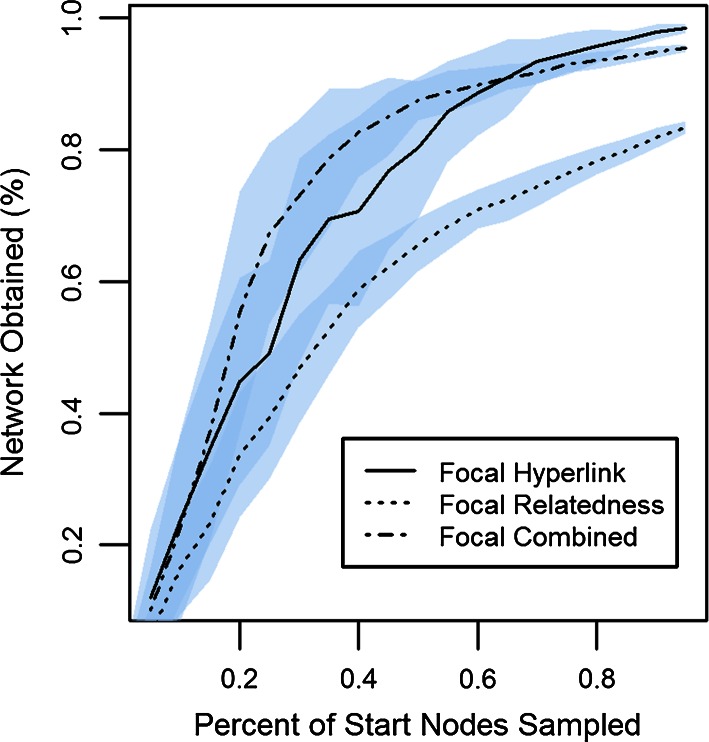
Plot of percent of start nodes used to initialize the network and the percent of the full network obtained after following snowballing procedures. The *shaded* cloud represents the standard deviation of the results for each sampled percentage

The sensitivity analysis indicated that the final focal networks were contingent on the identities and number of start nodes used to initialize the network (Fig. 3). When sampling a small percentage of start nodes (e.g., below 40 %), <60 % of the nodes in the final focal network were obtained. However, growth was rapid and reached 100 % of network obtained at approximately 65 % sampling of start nodes. The combined method increased slightly more rapidly than did the two methods independently.

## Discussion

### Summary of Results

The goal of the presented work was to introduce two internet-based methods for building social networks and contrast them with a traditional survey-based approach to SNA to facilitate interpretation of the differences among results.

The two web-based approaches resulted in much bigger networks than did the traditional approach, because we used multiple rounds of snowballing to grow the networks, while the traditional network was not snowballed. Also notably, the internet-based networks were much less connected than the survey-based network. The traditional network had an organizational response rate (69 %) well above the average survey response rate for organizations (36 %) (Baruch and Holtom 2008) and was particularly well connected with a relatively smooth cumulative frequency distribution, differing from the expected highly skewed or power law degree distributions (Fig. 2) (Clauset et al. 2009; Newman et al. 2002). However, when combined, the two internet-based approaches captured a large portion of the organizations identified in the traditional network. This indicates that network-based approaches might not capture as many relationships as the traditional approach but can be very useful for capturing a comprehensive list of stakeholders and identifying novel potential partners.

The lack of overlap in important nodes—those with the highest degree—between the internet-based and traditional networks could help conservation organizations identify partners that might play particularly useful roles in their network and that may be currently underutilized. For example, the internet-based approaches both had two universities in their top five most important nodes. These universities may be called upon to act as a bridge between disparate parts of the network by providing a good conduit for information sharing. Further it might be particularly useful for organizations to build formal partnerships with these organizations through, for example, collaboration on a grant or co-ownership of monitoring equipment.

In terms of organizational types represented in the networks, informational organizations were better represented in the internet-based networks than in the traditional network. It is logical that informational organizations have a strong web presence. They may not be considered “partners” in traditional networks, although they provide a very important function in a network. Identifying these informational organizations is especially useful if the purpose of building and analyzing the network is to help facilitate knowledge transfer.

There were also qualitative differences between the networks. Although we did not georeference the headquarters of the organizations in the internet-based networks, many were based outside the Greater Chicago area. This characteristic of internet-based networks may be detrimental for identifying only regional partners, but it also may identify potential partners working on similar conservation issues in different places.

### Advantages

The interpretation of any network is contingent upon the manner in which the links are defined. In traditional approaches, the definition of a link is very clear. For example, they are generally self-defined and based on specific criteria or questions in a survey [i.e., collaborators in the past 2 years (Vance-Borland and Holley 2011)]. Links identified through web-based approaches are less clearly defined, but the unique insights gained from these approaches provide significant advantages. Both hyperlink and relatedness methods of link definition have the potential to provide insight about their organization’s web presence, which has become increasingly important for conservation organizations.

### Hyperlink Method

The hyperlink method makes it possible for an organization to see how an Internet user might transverse their portion of the Web. It provides insight about the likelihood that someone exploring other stakeholders’ websites will find their website. This information might reveal how the organization can better position itself to direct more web traffic to their website. Additionally, although the meaning behind a hyperlink on a website could be interpreted many ways (i.e., a funding organization or a recent collaborator), it is self-defined as are the survey-based approaches.

### Relatedness Method

In contrast to the hyperlink method, the relatedness method explores the likelihood that a web browser (Google in particular) will find the organization’s website. Similar to the hyperlink approach, it represents a particular way that the public interacts with the Internet. This approach is not self-defined so a relatedness network is a network more of affinity than affiliation. Also, the meaning of the connection is less clear due to the proprietary nature of the algorithm that generates related sites. However, not being self-defined provides an outside perspective on connections and may identify novel links overlooked by other approaches.

### General Advantages

Internet-based approaches to SNA have numerous advantages for conservation organizations. Data for internet-based SNAs are free and publicly available. The only costs associated with developing the networks are the cost of staff time, which is required to develop the objectives, execute the methods, and conduct the network analysis. Growing web-based stakeholder networks by snowballing is relatively easy and straightforward. Snowballing of traditional survey-based networks drastically increases the time requirements for a project and thus may be too costly to conduct more than one or two rounds. Finally, combining several different internet-based methods, as we have done here, can compensate for the relative weaknesses of a single internet-based approach (see below) and further tease out pertinent information about the “true” social network. The Internet is constantly changing, so the results of web-based methods are unique to the time at which the analysis was conducted. Once the methods are established, organizations can easily monitor changes in their networks through time, which will provide feedback on the effectiveness of their efforts and identify new potential contacts or areas of common interest.

### Caveats and Limitations

Despite clear advantages to the web-based approaches, there are some important limitations. The biggest limitation is the level of technical understanding necessary to carry out the analyses. Currently, this requires programming skills, although a user-friendly interface could be developed, making the approach accessible to a much wider audience.

Also, these methods are restricted to the organizational level and not appropriate for analysis of an individual’s influence in the network, except in the case of individuals who have a very strong web presence, such as publicly elected officials. Also, the organization’s size can greatly impact how it is represented in the network. Very small organizations, regardless of its actual stakeholder network influence, may be nearly or completely missed in these approaches because they do not have or do not maintain a website. Also, organizations with less capacity might update the links on their site less frequently. Large organizations that have resources devoted to developing a web presence and content may have an inflated presence in these networks. Further, a very large corporation may have an international web presence but less clearly call out the activities of a branch or regional office that is most active in the conservation network. In other words, importance in an internet-based network may not directly correlate to importance in a stakeholder network.

Start nodes for an internet-based network have the potential to skew the network in an unanticipated manner. For example, our original network contained a start node for a small hobby-based club with only a peripheral interest in environmental issues. This resulted in an entire network component related to their hobby and unrelated to the Calumet environmental stakeholder network. Additionally, sensitivity analysis indicated that the final network was contingent on the list of start nodes for the internet-based networks. Therefore, it is important to put effort into the collection of the initial list. Further, because the internet-based methods are relatively easy to rerun, we suggest running a sensitivity analysis on networks derived through these methods to get a better understanding of the stochastic nature of networks and help determine if more effort is needed for generating the list of start nodes.

## Conclusions

There are several instances when conservation professionals should consider an internet-based approach to SNA. Ultimately, the web-based approaches do not provide an exact replacement for the traditional approach to SNAs. The information that they do provide—about electronic presence and a proxy but not a replacement for social relationships—is valuable in its own right. These methods may also serve as a complementary analysis to traditional approaches. Due to the numerous logistical benefits and information supplied, internet-based SNAs may provide conservation practitioners with a much needed and cost-effective tool, in analysis and intentional design of collaborative networks.
